# Supplementary material for: Sustainable polycarbonate adhesives for dry and aqueous conditions with thermoresponsive properties
Source: Nat Commun. 2019 Dec 2;10:5478. doi: 10.1038/s41467-019-13449-y (PMC6889139; doi:10.1038/s41467-019-13449-y)
Supplement: Supplementary file 1 — Supplementary Information [file 41467_2019_13449_MOESM1_ESM.pdf]

Supplementary Information

Sustainable Polycarbonate Adhesives for Dry and Aqueous Conditions with Thermo-responsive Properties

Anjeza Beharaj, Ethan Z. McCaslin, William A. Blessing, and Mark W. Grinstaff*

Supplementary Methods

Epoxide Monomer. Glycidyl butyrate was synthesized according to previous literature.[1] A roundbottom flask was charged with 300 ml dichloromethane, trimethylamine (84 mL, 600 mmol) and glycidol (20 mL, 300 mmol). The reaction was placed in an icebath and stirred for 30 minutes. Butyryl chloride (35 mL, 333 mmol) was added dropwise and the reaction was stirred for another 3 hours at room temperature. The subsequent solution was filtered to remove the salt precipitate. The organic layer was washed with saturated sodium bicarbonate (300 mL) three times, once with brine (300 mL), dried over sodium sulfate, and evaporated off under low pressure. The crude yellow product was fraction distilled under vacuum at 110 ^{o}C to produce pure glycidyl butyrate as a clear oil. (33 mL, 229 mmol, 76% yield).

Catalyst. The salen ligand cobalt catalyst as synthesized as previously described by Lu *et al.*[2,3]

Polymer Synthesis. In a glovebox, glycidyl butyrate (0.67 mL, 5 mmol) propylene oxide (0.35 mL, 5 mmol) were added into a high pressure autoclave, followed by the addition of *(S,S)*-SalcyCo^{III}DNP (5.21 mg, 0.005 mmol). The autoclave was transferred out of the glovebox and charged with CO_2 to 2.7 MPa. The reaction was allowed to run at 40 °C for 10 hr. Subsequently, the reaction vessel was placed in an ice bath for 10 minutes and the CO_2 pressure was released. The reaction mixture was diluted in minimal amount DCM. The mixture was added dropwise into cold MeOH (50 ml) and the precipitated polymer was collected. The precipitation was repeated for a total of 3 times until complete removal of the catalyst and unreacted monomer. The resultant material was dried under vacuum to yield ~ 300 mg of a viscous liquid.

Supplementary Tables

entry	$f_{[GB]}$	$f_{[PO]}$	$F_{[GB]}$	$F_{[PO]}$	Time (h)	Conversion %	X	Y	H	G
1	0.9	0.1	0.93	0.07	1	5.7	9	12.5	6.48	8.28
2	0.7	0.3	0.79	0.21	1	7.6	2.333	3.846	1.415	1.726
3	0.5	0.5	0.63	0.37	1	12	1	1.73	0.578	0.421
4	0.3	0.7	0.47	0.53	1	9	0.428	0.877	0.209	-0.06
5	0.1	0.9	0.28	0.72	1	6.6	0.111	0.392	0.031	-0.17222

Supplementary Table 1. Fineman-Ross table of PPGBC at low conversions. F = molar ratio of monomer. F= molar ratio of monomer incorporation in polymer. X= [$f_{[GB]}$/ $f_{[PO]}$, Y= [$F_{[GB]}$/ $F_{[PO]}$], H= Y^2/X, G=Y(X-1)/X.

Supplementary Figures

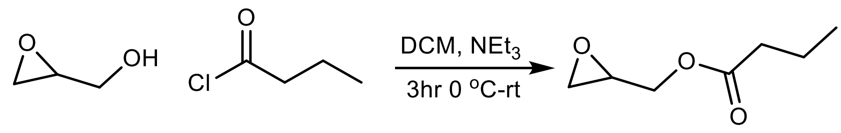

Supplementary Figure 1. Synthetic pathway of glycidyl butyrate from glycidol and butyryl chloride.

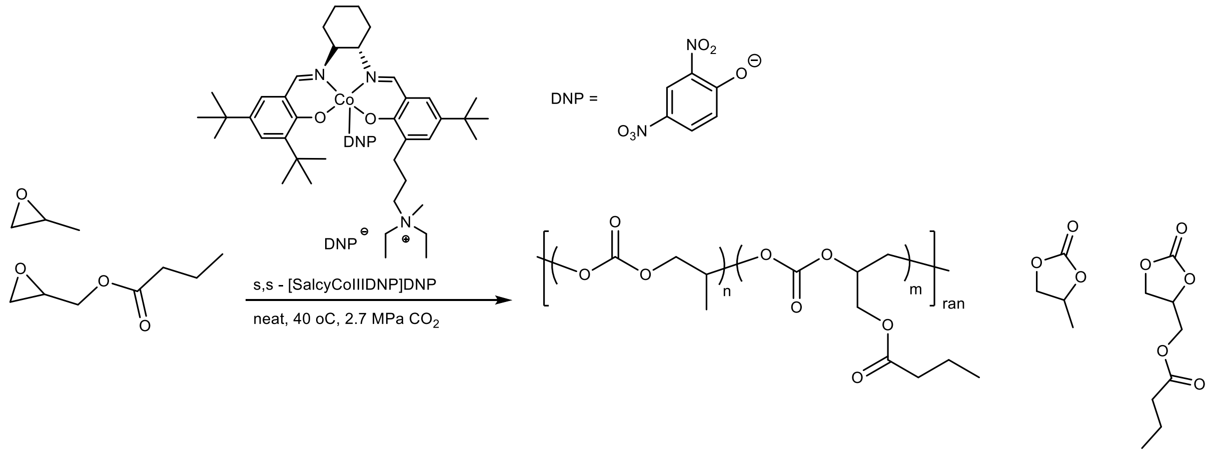

Supplementary Figure 2. Synthesis of polycarbonate terpolymers showing a representative procedure for terpolymerization of oxiranyl ester with CO_2 using [*S,S*]-[SalcyCo^{III}DNP]/DNP.

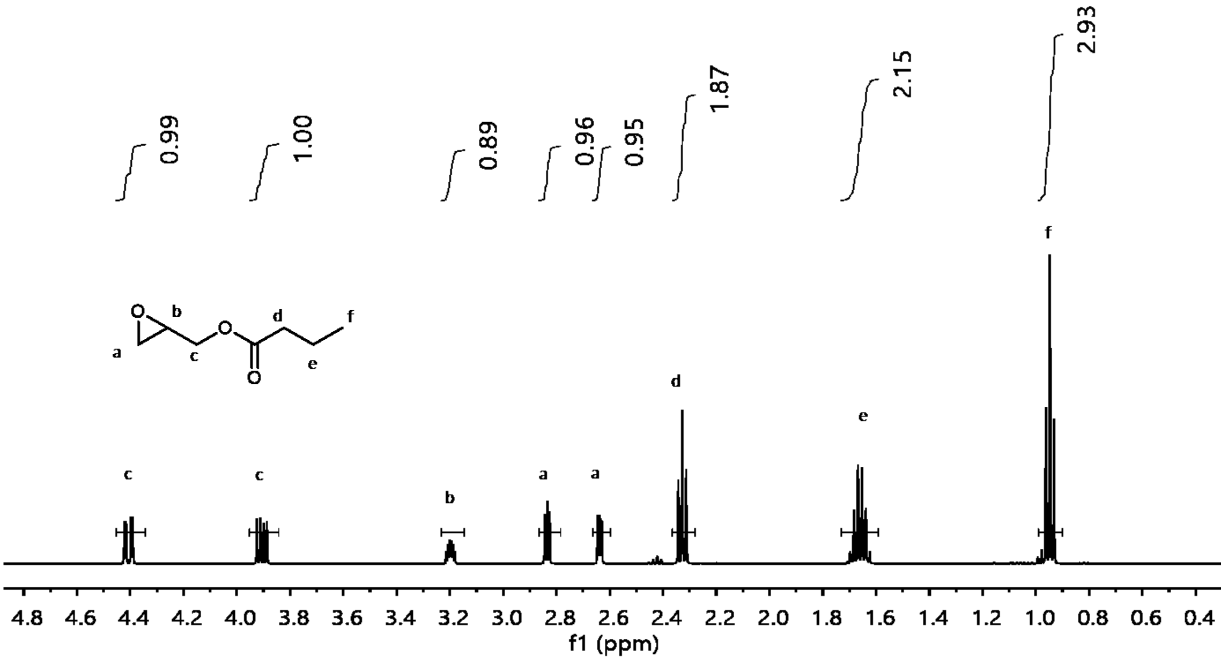

Supplementary Figure 3. ^{1}H NMR spectrum of glycidyl butyrate in $CDCl_3$.

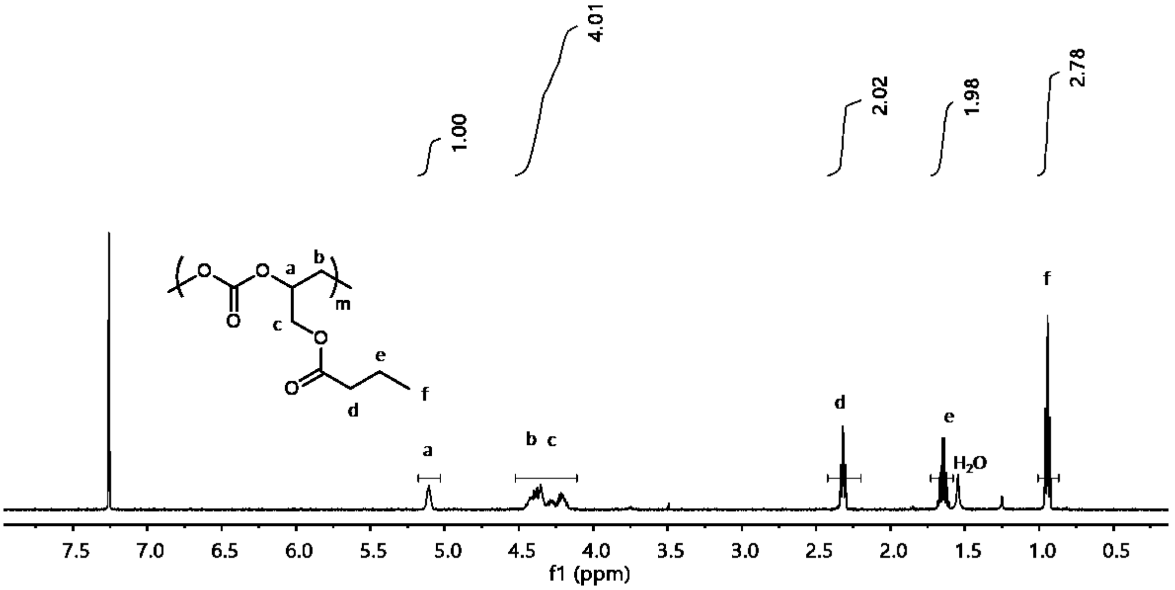

Supplementary Figure 4. ^{1}H NMR spectrum of poly(glycidyl butyrate carbonate) in $CDCl_3$. Additional characterization can be found in supplementary references.[4]

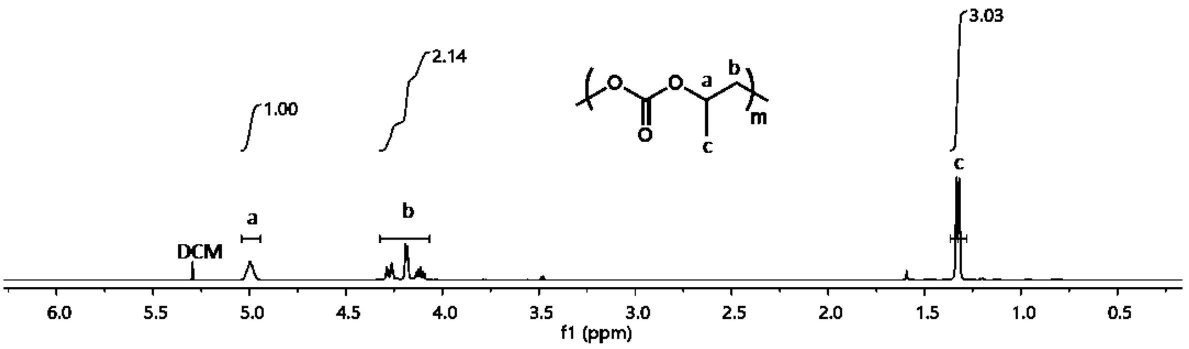

Supplementary Figure 5. ^{1}H NMR spectrum of poly(propylene carbonate) in $CDCl_3$. Additional characterization can be found in supplementary references.[5]

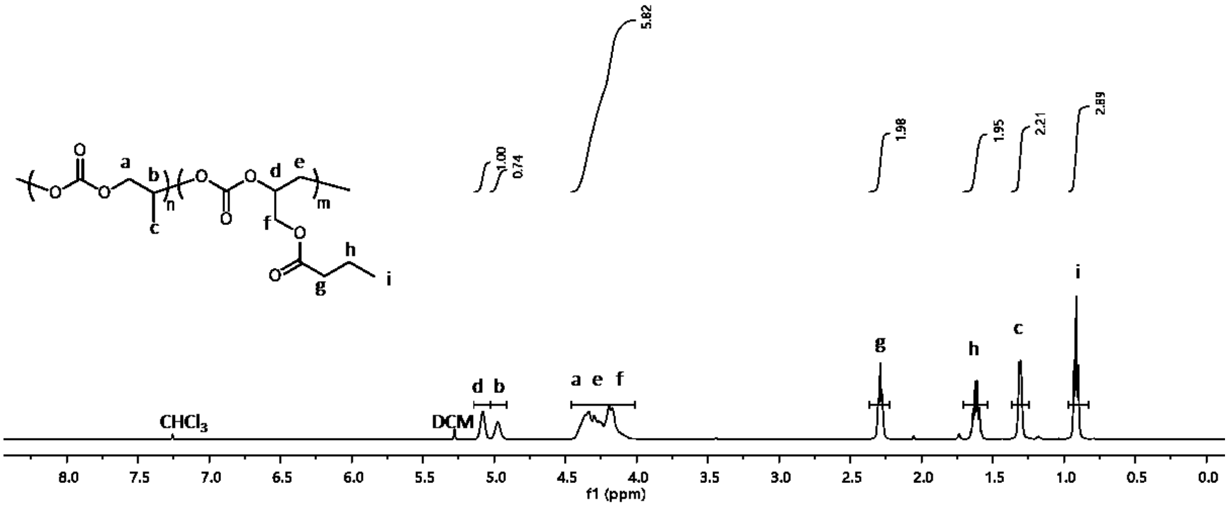

Supplementary Figure 6. ^{1}H NMR spectrum of poly(propylene-*co*-glycidyl butyrate carbonate) in $CDCl_3$.

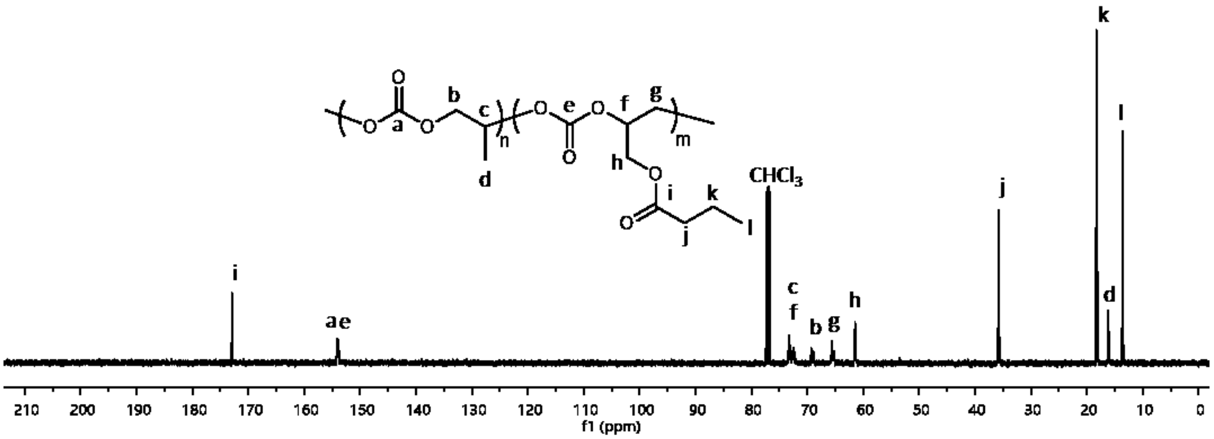

Supplementary Figure 7. ^{13}C NMR spectrum of poly(propylene-*co*-glycidyl butyrate carbonate) in $CDCl_3$.

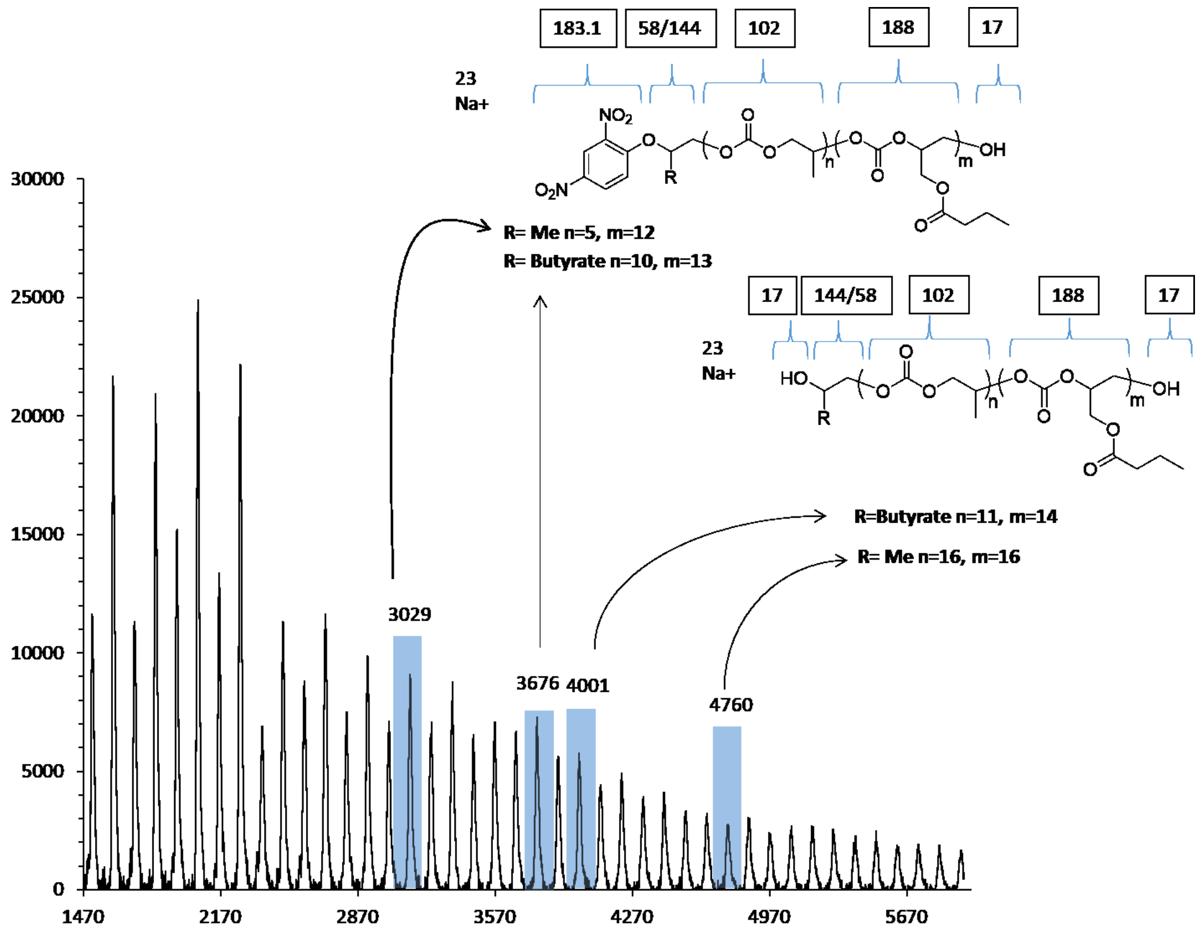

Supplementary Figure 8. A MALDI-TOF spectrum of PPGBC-(56).

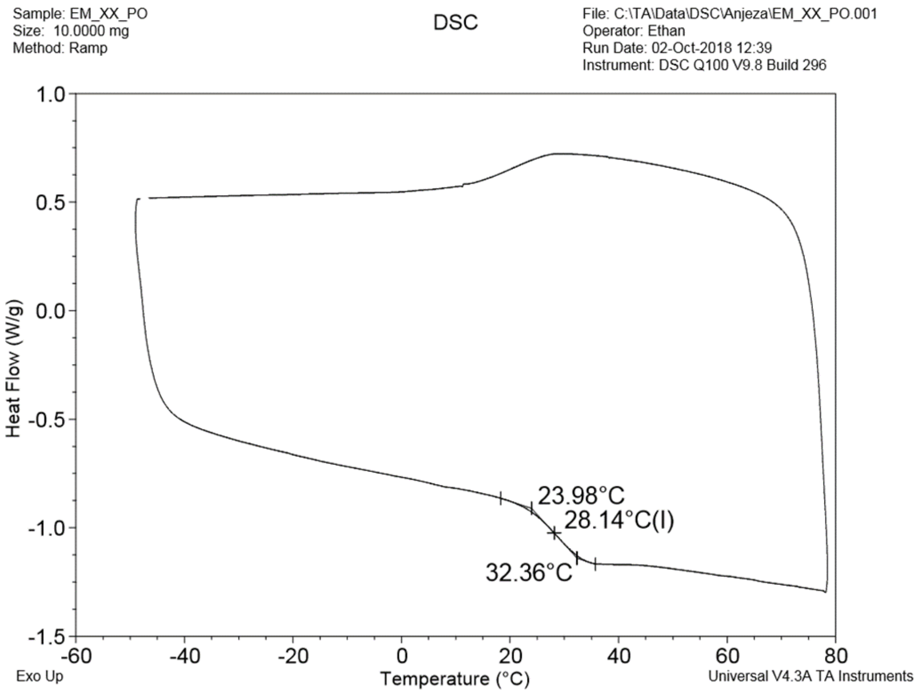

Supplementary Figure 9. DSC thermogram of poly(propylene carbonate).

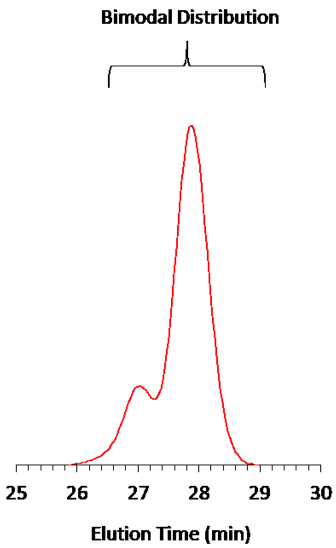

Supplementary Figure 10. GPC trace of poly(propylene-*co*-glycidyl butyrate carbonate). All polymers exhibited bimodal distribution peaks.

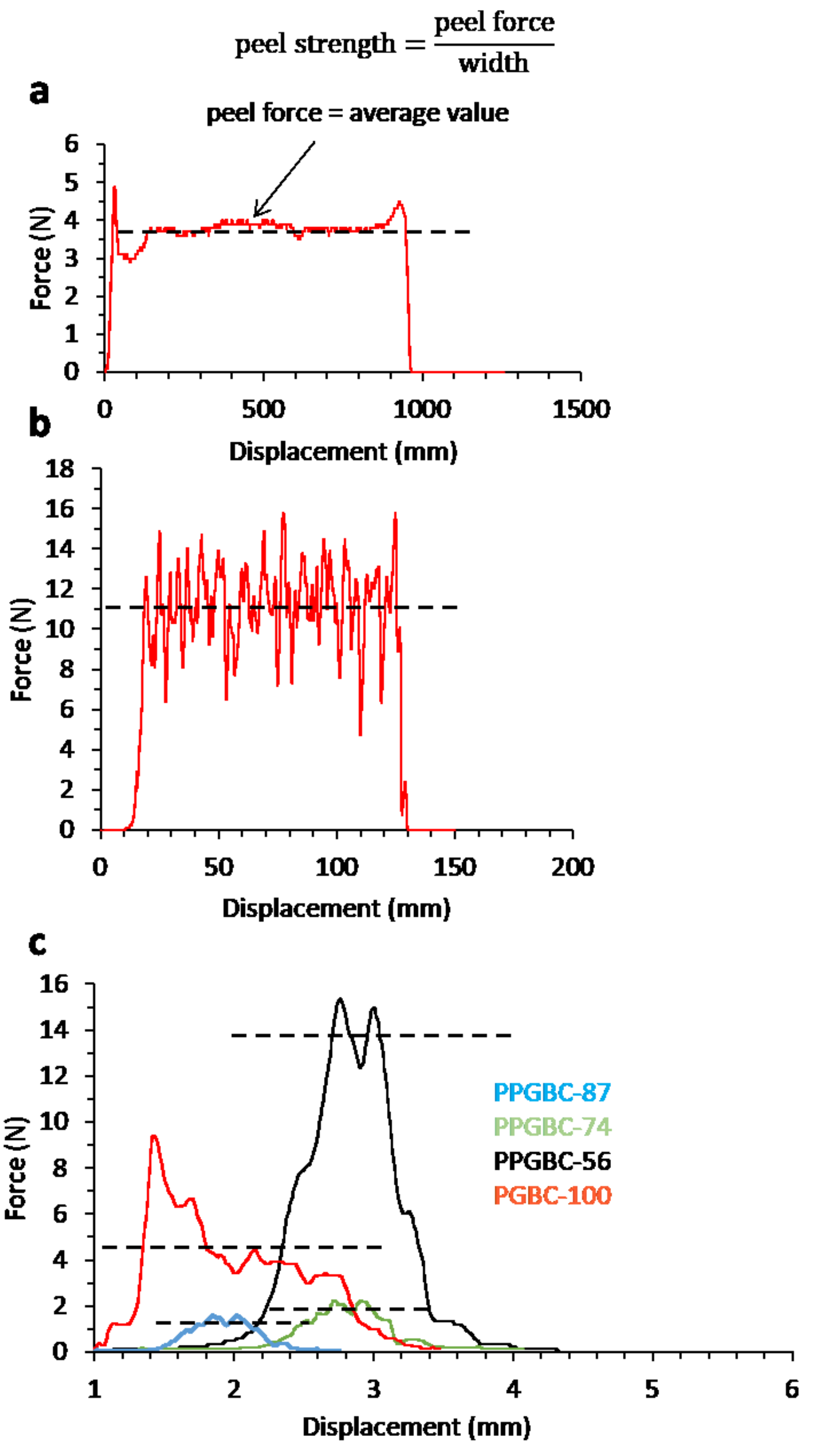

Supplementary Figure 11. Instron 180° peel traces of **a.)** Scotch Tape ® (N=3) **b.)** Duct Tape ® (N=3). **c.)** room temperature (22 °C) viscous poly(propylene-*co*-glycidyl butyrate carbonate) polymers (N=3).

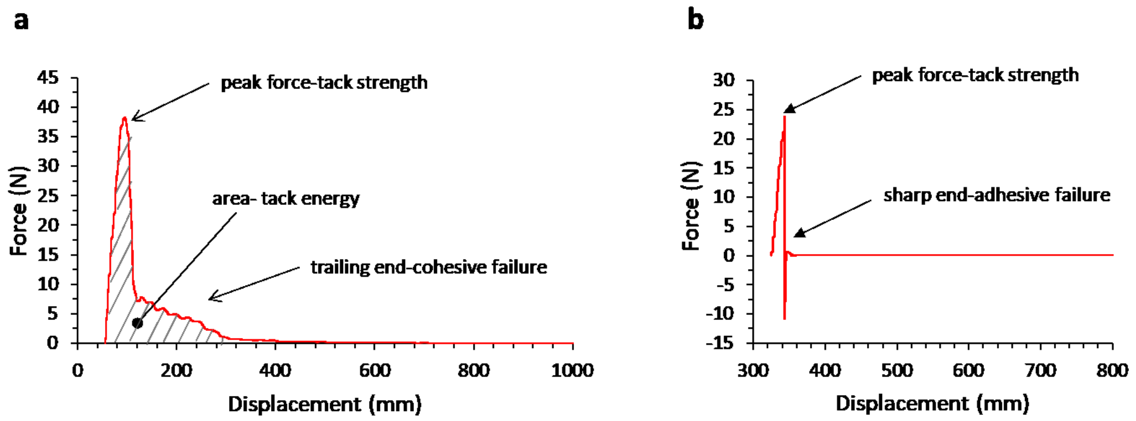

Supplementary Figure 12. Representative tack trace of carbonate polymers in DHR-2 rheometer**. a** PPGBC-56 at 25 °C and 50 N applied axial force. **b** PPGBC-56 at 20 °C and 20 N applied axial force.

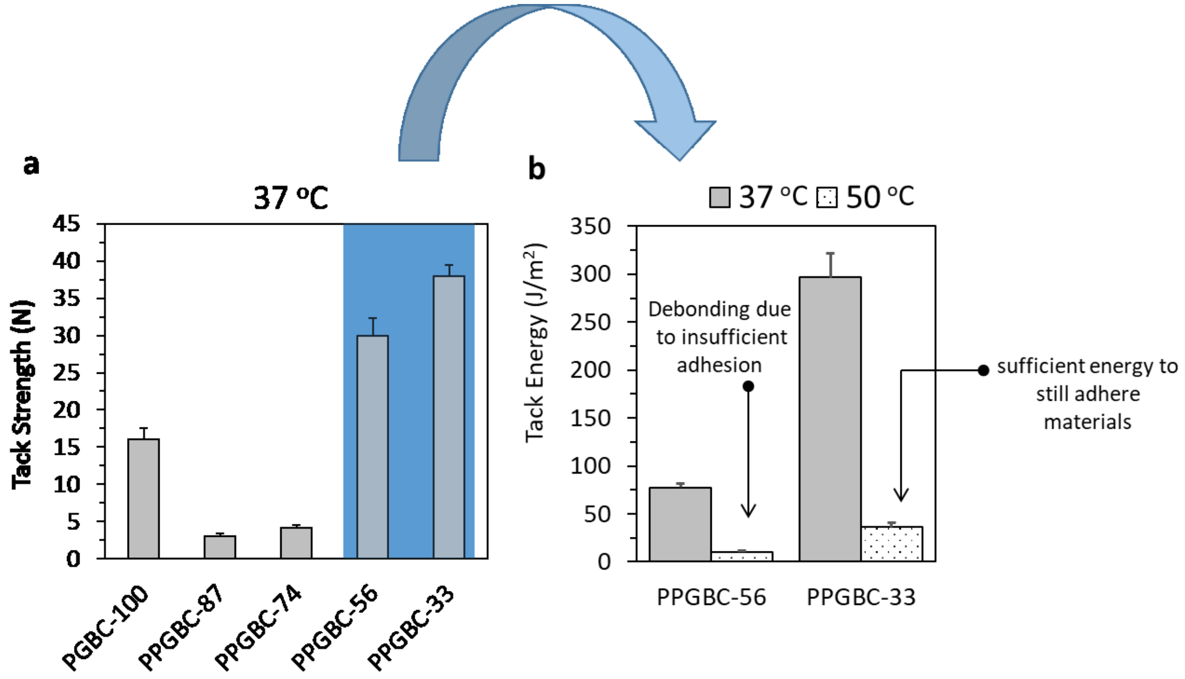

Supplementary Figure 13. Tack strength of PPGBC polymers at 37 °C. **a** PPGBC-33 and PPGBC-56 exhibit the highest tack strength (n=3). **b.** Tack energy of PPGBC-56 and PPGBC-33 at 37 °C and 50 °C (n=3). PPGBC-56 exhibits weaker adhesion and thermal properties than PPGBC-33, though glass transition temperatures are similar. Terpolymer PPGBC-22 was also tested at 25 °C, 37 °C, and 50 °C, but exhibited a tack strength higher than the upper limit of the machine's detection (> 55N). Error bars indicate mean ± s.e.m.

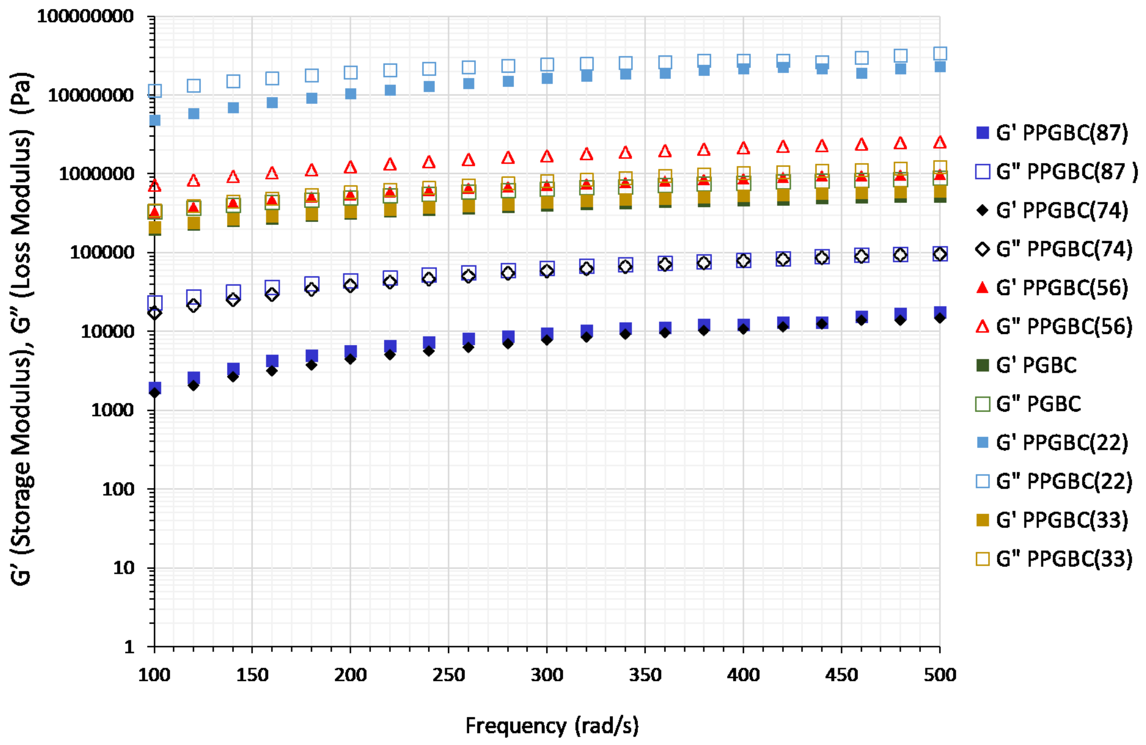

Supplementary Figure 14. Storage (G') and loss (G") modulus for PPGBC polymers at high frequency corresponding to peel strength.

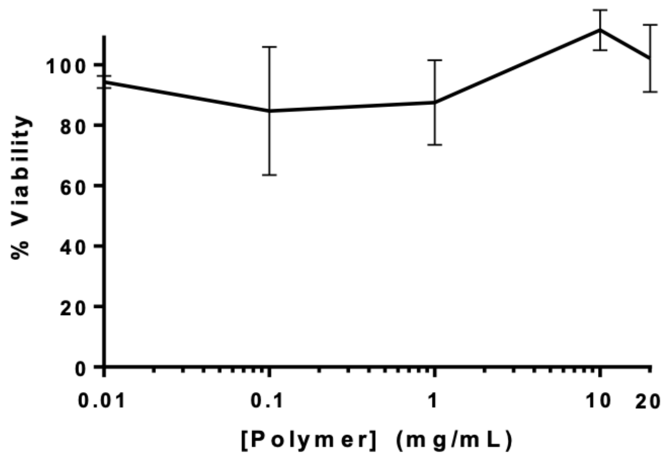

Supplementary Figure 15. Cytotoxicity to NIH 3T3 fibroblasts of PPGBC-56 at various concentrations. Any toxicity observed is independent of dose, suggesting limited PPGBC-56 cytotoxicity.

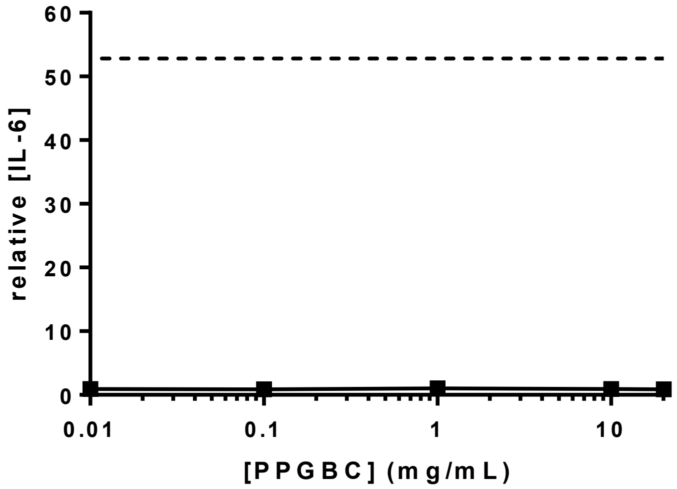

Supplementary Figure 16. Immunogenicity of PPGBC-56 to RAW 264.7 macrophages at various concentrations. IL-6 is not upregulated at any concentration of polymer and shows no *in vitro* immunogenicity. The dashed line represents relative expression of IL-6 with lipopolysaccharide treatment.

Supplementary References

1. Adilson, B. *et al.* Synthesis and enzymatic resolution of racemic 2,3-epoxy propyl esters obtained from glycerol. *Tet. Lett.* **56,** 1696–1698 (2015).

2. Lu, X.B. *et al.* Highly active, bifunctional Co(III)-salen catalyst for alternating copolymerization of CO_2 with cyclohexene oxide and terpolymerization with aliphatic epoxides. *Macromolecules* **43,** 1396-1402 (2010).

3. Lu, X.B. *et al.* Mechanistic aspects of the copolymerization of CO_2 with epoxides using a thermally stable single-site cobalt(III) catalyst. *J. Am. Chem. Soc.* **131,** 11509–11518 (2009).

4. Beharaj, A., Ekaldious, I. & Grinsaff, M.W. Poly(alkyl glycidate carbonate)s as degradable pressure-sensitive adhesives. *Angew. Chem., Int. Ed.* **131 (5),** 1421-1425 (2019).

5. Cohen, C.T., Chu T. & Coates, G. W. Cobalt catalysts for the alternating copolymerization of propylene oxide and carbon dioxide: combining high activity and selectivity. *J. Am. Chem. Soc.* **127 (31),** 10869-10878 (2005).
