# Supplementary material for: Sustainable polycarbonate adhesives for dry and aqueous conditions with thermoresponsive properties
Source: Nat Commun. 2019 Dec 2;10:5478. doi: 10.1038/s41467-019-13449-y (PMC6889139; doi:10.1038/s41467-019-13449-y)
Supplement: Supplementary file 2 — Description of Additional Supplementary Files [file 41467_2019_13449_MOESM2_ESM.docx]

Description of Additional Supplementary Files

**Supplementary Movie 1:** Adhesion Potential of PPGBC-56 of a glass cube and metal rod at 21^o^C under water. Contact time is 5 seconds; contact area is 56 mm^2^ ; applied axial force is non other than the weight of the rod (35g). The glass cube has a weight of 25g.

**Supplementary Movie 2:** Adhesion Potential of PPGBC-56 of a glass cube and metal rod at 37^o^C under water. Contact time is 5 seconds; contact area is 56 mm^2^ ; applied axial force is non other than the weight of the rod (35g). The glass cube has a weight of 25g.

**Supplementary Movie 3:** Adhesion Potential of PPGBC-56 of a glass cube and metal rod at 50^o^C under water. Contact time is 5 seconds; contact area is 56 mm^2^ ; applied axial force is non other than the weight of the rod (35g). The glass cube has a weight of 25g.
